# Dendritic spinule-mediated structural synaptic plasticity: Implications for development, aging, and psychiatric disease

**DOI:** 10.3389/fnmol.2023.1059730

**Published:** 2023-01-20

**Authors:** Colleen R. Zaccard, Isabel Gippo, Amy Song, Changiz Geula, Peter Penzes

**Affiliations:** ^1^Department of Neuroscience, Northwestern University Feinberg School of Medicine, Chicago, IL, United States; ^2^Mesulam Center for Cognitive Neurology and Alzheimer's Disease, Northwestern University Feinberg School of Medicine, Chicago, IL, United States; ^3^Department of Psychiatry and Behavioral Sciences, Northwestern University Feinberg School of Medicine, Chicago, IL, United States

**Keywords:** dendritic spines, memory and cognition, synaptic activity, synaptic connectivity, electron microscopy, enhanced resolution microscopy, neurodegenerative disease, postsynaptic density

## Abstract

Dendritic spines are highly dynamic and changes in their density, size, and shape underlie structural synaptic plasticity in cognition and memory. Fine membranous protrusions of spines, termed dendritic spinules, can contact neighboring neurons or glial cells and are positively regulated by neuronal activity. Spinules are thinner than filopodia, variable in length, and often emerge from large mushroom spines. Due to their nanoscale, spinules have frequently been overlooked in diffraction-limited microscopy datasets. Until recently, our knowledge of spinules has been interpreted largely from single snapshots in time captured by electron microscopy. We summarize herein the current knowledge about the molecular mechanisms of spinule formation. Additionally, we discuss possible spinule functions in structural synaptic plasticity in the context of development, adulthood, aging, and psychiatric disorders. The literature collectively implicates spinules as a mode of structural synaptic plasticity and suggests the existence of morphologically and functionally distinct spinule subsets. A recent time-lapse, enhanced resolution imaging study demonstrated that the majority of spinules are small, short-lived, and dynamic, potentially exploring their environment or mediating retrograde signaling and membrane remodeling *via* trans-endocytosis. A subset of activity-enhanced, elongated, long-lived spinules is associated with complex PSDs, and preferentially contacts adjacent axonal boutons not presynaptic to the spine head. Hence, long-lived spinules can form secondary synapses with the potential to alter synaptic connectivity. Published studies further suggest that decreased spinules are associated with impaired synaptic plasticity and intellectual disability, while increased spinules are linked to hyperexcitability and neurodegenerative diseases. In summary, the literature indicates that spinules mediate structural synaptic plasticity and perturbations in spinules can contribute to synaptic dysfunction and psychiatric disease. Additional studies would be beneficial to further delineate the molecular mechanisms of spinule formation and determine the exact role of spinules in development, adulthood, aging, and psychiatric disorders.

## 1. Introduction

Dendritic spines are small, actin-rich, membrane protrusions of neuronal dendrites that form functional contacts with nearby axonal boutons to receive electrical or chemical input signals (Hering and Sheng, 2001). Spines represent a specialized compartment for most excitatory synaptic transmission in the mature mammalian brain, with a mature spine typically forming a single synapse at its head (Hering and Sheng, 2001). While spines are often 0.5–2 microns in length, they can extend up to 6 microns, and larger spines contain more diverse organelles and larger synapses (Harris and Kater, 1994). Their composition includes an electron-dense thickening of the postsynaptic membrane at the synaptic junction, termed the postsynaptic density (PSD), which contains many proteins involved in the regulation of synaptic function (Sheng and Kim, 2011). The PSD is aligned with the presynaptic active zone and can be continuous and simple or discontinuous and complex in shape, i.e., fenestrated, horseshoe, perforated, or segmented (Hering and Sheng, 2001). Preceding the formation of spines during early development, neuronal dendrites extend long, thin processes, termed dendritic filopodia, which are highly dynamic and transient (Fiala et al., 1998). Dendritic filopodia sample their environment for new axonal partners and can facilitate synaptogenesis by initiating contact with presynaptic terminals, eventually transitioning into spines (Fiala et al., 1998; Mao et al., 2018). Four basic classes of spines have been proposed: Thin, stubby, mushroom, and branched (Harris et al., 1992). Thin spines concentrate calcium ions (Ca^2+^) and respond to rapid changes in synaptic activity, suggesting they are “learning” spines, while more mature and stable mushroom spines exhibit greater complexity in their molecular architecture and are thought of as “memory spines” (Bourne and Harris, 2007).

Synaptic plasticity underlies cognitive processes and memory and can be mediated by functional alterations at synapses, i.e., changes in synaptic strength, or structural changes at synapses (Citri and Malenka, 2008). Spines are highly dynamic, and modifications in their density, shape, and/or size serve as the basis for structural synaptic plasticity (Hering and Sheng, 2001; Holtmaat and Svoboda, 2009; Kasai et al., 2010). It has been postulated that intrinsic fluctuations in spine volume are necessary for synapse maintenance over time, while fast synaptic activity-induced plasticity is important for cognitive processes (Kasai et al., 2010). Structural spine dynamics can mediate short- and long-term plasticity at excitatory synapses, and result in changes to local network organization (Kasai et al., 2010). Impairments in structural synaptic plasticity play a role in major psychiatric disorders, including intellectual disability, schizophrenia, and autism spectrum disorder (Citri and Malenka, 2008; Bernardinelli et al., 2014). Yet, the mechanisms underlying experience-dependent structural plasticity in cognition are not fully understood.

This review focuses on fine membranous protrusions at synapses, termed spinules, with an emphasis on the most widely studied spinule type, dendritic spinules, which emerge from spines. Spinules can invaginate nearby spines, axonal boutons, or glial cells, and potentially serve as a mode of localized communication to mediate structural synaptic plasticity (Petralia et al., 2015, 2018). Spinules can originate from postsynaptic spines, presynaptic terminals, or glial cell projections, and the range of small invaginating projections has been reviewed extensively (Petralia et al., 2015, 2017). While invaginating projections are present in invertebrates, such as cnidarians (Westfall, 1970; Case et al., 1972), they appear to have evolved in complexity in mammals that demonstrate a greater degree of synaptic plasticity (Petralia et al., 2015, 2018). Dendritic spinules, referred to herein simply as “spinules,” are finer than filopodia, variable in length, often emerge from large mushroom spines, and can be positively regulated by synaptic activity (Petralia et al., 2015). In a 1962 study by Westrum and Blackstad (1962) of the adult rat hippocampal stratum radiatum, spinules were first observed projecting from pyramidal cell spines and invaginating presynaptic terminal membranes. The tips of spinules are often surrounded by clathrin-coated pits at the end of presynaptic membrane invaginations, indicating a potential role in neuronal communication (Westrum and Blackstad, 1962; Tarrant and Routtenberg, 1977). An increase in spinule density following the induction of long-term potentiation (LTP) has been well documented in the rat dentate gyrus (Schuster et al., 1990), and an increase in spinule length and size has similarly been reported in rat hippocampal organotypic cultures (Toni et al., 1999). Since their discovery, spinules have been observed in the hippocampus, cerebellum, and cerebral cortex (Petralia et al., 2015). An EM study of the basal state adult rat hippocampus revealed that 91% of mushroom spines and 24% of thin spines form one or more spinules (Spacek and Harris, 2004), implying that spinules occur frequently in the mature healthy brain. Spinules can arise from perforations or edges of complex PSDs (Toni et al., 2001; Dhanrajan et al., 2004; Spacek and Harris, 2004), or emerge from non-perforated PSD edges or spine necks (Sorra et al., 1998; Tao-Cheng et al., 2009). The observed differences in spinules in relation to PSD complexity suggest the possibility of structurally distinct spinule subtypes, necessitating further investigation.

The diffraction limits of light microscopy techniques have hindered researchers ability to assess nanoscale spinules in experimental datasets of spines (Zaccard et al., 2021). In one key investigation of spinules in dissociated hippocampal rat neurons, ~70% of spines that appeared to be globular or cup shaped at low resolution were revealed to be more complex structures composed of multiple spinule-like protrusions at super-resolution (Chazeau et al., 2014). Because of these limitations, much of our knowledge of spinule structure, regulation, and function has been deduced from single snapshots in time acquired using electron microscopy (EM) of fixed samples. However, recent advances in enhanced resolution confocal imaging techniques have yielded new insights into spinule dynamics, regulation, and functions (Zaccard et al., 2020, 2021). Here we summarize the current knowledge about the molecular mechanisms of mammalian spinule formation. We discuss investigations of spinules in development, adulthood, and aging, highlighting recent investigations that reveal surprising structural and functional diversity of spinules. Finally, we survey the evidence for underlying changes in spinules in psychiatric disorders, including intellectual disability and neurodegenerative diseases.

## 2. Mechanisms of spinule formation

Numerous studies have linked spinule formation to neuronal activity and structural LTP. In hippocampal slice cultures, EM revealed spinules 80–500 nm in length with varying morphologies that were induced by high potassium (K^+^) depolarization and N-methyl-D-aspartate (NMDA) treatment (Tao-Cheng et al., 2009). Similarly, spinules have been induced by theta burst stimulation for the induction of LTP (Toni et al., 1999). Live confocal imaging of hippocampal slice cultures revealed long spinule-like protrusions of spines induced by exogenous glutamate application, with orientation directed toward the glutamate (Richards et al., 2005). Further, LTP induction by two-photon glutamate uncaging can induce spinule formation (Ueda and Hayashi, 2013).

Published studies implicate central signaling molecules and pathways underlying synaptic transmission and plasticity in spinule formation. Ca^2+^ is a key signaling ion in synaptic transmission and regulates spinule-dependent synaptic plasticity in fish horizontal cells (Country and Jonz, 2017). Actin regulatory pathways may be involved in spinule formation, as one study noted F-actin-driven spinules induced by electrical stimulation (Colicos et al., 2001), and another study described “spine head protrusions” formed by Rac1-dependent elongation of F-actin barbed ends away from the PSD (Chazeau et al., 2014). The lipid second messenger, phosphatidylinositol-3,4,5-trisphosphate (PIP_3_), supports a range of neuronal functions, including maintenance of AMPA receptor clustering during LTP (Arendt et al., 2010). Local PIP_3_ has been shown to positively regulate spinules during structural LTP (Ueda and Hayashi, 2013). Kalirin, a guanine nucleotide exchange factor, plays an essential role in structural spine plasticity downstream of NMDA receptors by inducing Ca^2+^/calmodulin-dependent protein kinase II (CaMKII)-mediated activation of the Rho-GTPase Rac-1, which in turn mediates actin polymerization and spine head enlargement (Xie et al., 2007). Importantly, the Sec14p-like domain of kalirin, which contains the CaMKII phosphorylation site, binds directly to PIP_3_ (Miller et al., 2015). Studies show that kalirin is essential for activity-dependent synaptic plasticity, as well as learning and memory (Ma et al., 2001; Xie et al., 2007).

We recently investigated the relationship between spinules and the PSD, and the roles of local Ca^2+^ and kalirin-7, the most common isoform in the adult brain, during spinule formation in dissociated cortical pyramidal mouse neurons (Zaccard et al., 2020). We used live, time-lapse, rapid structured illumination microscopy and enhanced resolution confocal microscopy to show that smaller, dynamic, short-lived spinules (<60 s) emerged from near the edges of simple, discrete PSDs of mushroom spines, while more stable, long-lived spinules (≥60 s) were associated with complex PSDs. Our results indicated that Ca^2+^ is essential for spinule formation and that Ca^2+^ nanodomains localize to long-lived spinules, typically isolated from, but in synchrony with spine head Ca^2+^ transients. Interestingly, spines with frequent high-amplitude Ca^2+^ transients displayed both short-lived and long-lived spinules, while spines with sporadic peaks developed only short-lived spinules. Short-lived spinule number per spine was positively correlated with Ca^2+^ peak mean and maxima, while long-lived spinule number was correlated with Ca^2+^ peak frequency and duration. Further, kalirin-7 promoted spinule formation, elongation, and recurrence, which was defined as the appearance, disappearance, and reappearance of a spinule at one topographical spine head location. Although kalirin-independent mechanisms likely exist, we hypothesize that kalirin-7 modulates activity-dependent spinule formation downstream of PIP_3_ and Ca^2+^ signaling, and that perturbations in this pathway contribute to spinule dysregulation and disease.

## 3. Emerging functions of spinules in localized structural synaptic plasticity

### 3.1. Spinules may be trans-endocytosed or participate in synapse formation and maintenance

Many EM studies have reported spinules invaginating presynaptic terminals, and their proposed functions include facilitating communication with opposing presynaptic terminals, the formation of new synapses, and increasing the stability or complexity of existing synapses. Here, we focus on the role of spinules in synapse formation and stability, as well as consider evidence for morphologically and functionally distinct spinule subsets. For reference, we compiled a table of key mammalian spinule studies that includes spinule origination and contacts, the mode of induction or developmental state, spinule morphology, and proposed spinule functions (Table 1). A serial section EM study of the developing rat hippocampus at postnatal day 15 revealed that the majority of spinules emerge from the edges of nonperforated PSDs, or from spine necks, and extend into distal axonal boutons (Sorra et al., 1998). A similar study of the adult male rat hippocampus showed that 84% of the spinules emerging from mushroom spines are engulfed by presynaptic axons and 16% by neighboring axons, while only 17% of spinules from thin spines are engulfed by presynaptic axons, 67% by neighboring axons, and 14% by astrocytic processes (Spacek and Harris, 2004). The presence of coated pits on the engulfing membrane at most spinule tips and the presence of double-membrane structures in the cytoplasm of axons and astrocytes suggests postsynaptic membrane remodeling and retrograde signaling *via* trans-endocytosis. Another investigation described the outgrowth of fine membrane projections toward nascent presynaptic actin puncta, implicating spinules in new synapse formation (Colicos et al., 2001).

**Table 1:** Investigations of mammalian spinule structure, induction, and possible functions.

Author, journal, year	Spinule origination and contacts	Cell type, brain region, and species	Mode of induction or developmental state	Microscopy technique and spinule morphology	Proposed spinule functions
Westrum and Blackstad, J Comp Neurol, 1962	Postsynaptic projections from the head or stalk of a spine invaginated the presynaptic terminal membrane	Hippocampal stratum radiatum of adult rats	Adulthood	EM; Spinule lengths from 75 to 150 nm and widths from 25 to 100 nm; Narrow, flattened, or leaflike in shape; Continuous with spines, except for very small spines	A feature of Grey type 1 synapses, suggestive of some relationship to impulse transmission
Tarrant and Routtenberg, Tissue Cell, 1977	Postsynaptic membrane projections invaginated presynaptic axonal membranes; Surrounded by presynaptic vesicles in the presynaptic terminal	Hippocampal dentate gyrus and caudate nucleus of adult rats	Adulthood; Spinules occurred within asymmetric synapses; 10.1% of hippocampal synapses contained spinules; 8.5% of caudate nucleus synapses contained spinules	EM; Spherical double membrane structures in cross section; Size varied from simple and shallow to deep and branched with coated vesicles	Material transport between presynaptic with postsynaptic processes and information transmission between neurons
Desmond and Levy, Brain Research, 1983	Postsynaptic structures from concave (cup-shaped) spines invaginated presynaptic terminals of asymmetric synapses	Hippocampal dentate gyrus molecular layer of adult male rats	<13% of the total number of synapses contained spinules in controls; High-frequency stimulation increased number of concave spines	EM; Some concave spine heads bore spinules and were shaped like a W, often with a split PSD	Enhanced synaptic functions
Schuster et al., Brain Res, 1990	Postsynaptic spinules found at axospinous synapses	Hippocampal CA3, CA4, and dentate gyrus of adult rats	Increased density of axospinous synapses containing spinules in the inner molecular layer following LTP *via* stimulation electrode, implanted stereotactically	EM; Postsynaptic membrane protrusions ran parallel to presynaptic invaginations; Occurred in active zones	Synapse modification *via* enhanced synaptic efficacy, or trans-endocytosis of postsynaptic membrane or other cargoes for recycling or communication
Jones and Calverley, Brain Res, 1991	Postsynaptic spinules associated with perforated synapses; Large spinules projected into presynaptic terminals	Parietal cortex of rats	Perforated synapses were assessed at 9 ages, from 0.5 months (m) to 22 m, i.e., from youth to old-age; Spinules most prominent in middle age at 12 m	EM; Small spinules noted in young rats (0.5 and 1 m), Short broad spinules in adulthood (7 and 10 m); Large spinules peaked in middle age (12 m) and decreased in old age (18 and 22 m)	A feature of perforated spines that may function in maintenance of PSD surface area
Sorra et al., J Comp Neurol, 1998	Postsynaptic; Originated from edges of nonperforated PSDs or from spine necks; Extended into distal axonal boutons	Developing hippocampus of rats	Development (postnatal day 15)	EM; Projections from spines extended from perforations in PSDs	Synaptic remodeling, formation, and stability; Enhance synaptic transmission
Toni et al., J Neurosci, 2001	Extensions from the postsynaptic membrane into the presynaptic ending	Hippocampal CA1 stratum radiatum of rats	Induction of LTP increased enlarged synapses with segmented and partitioned PSDs, typically containing coated vesicles and large PSD-associated spinules	EM; Small, finger like protrusions were classified as small if shorter than 0.2 μm and large if longer than 0.2 μm	Synaptic remodeling; May underlie the formation of synapses with perforated PSDs
Colicos et al., Cell, 2001	Postsynaptic actin sprouted projections toward new presynaptic actin puncta, resembling axon-dendrite interactions during synaptogenesis	Dissociated hippocampal neurons of rats	Targeted photoconductive stimulation to elicit neuronal excitation; Single tetanus elicited reversable remodeling; Repeated tetanus trains induced stable remodeling and small postsynaptic projections	Live video imaging using standard light microscopy; Small projections	Postsynaptic actin spinules appear to contact the presynaptic actin condensation points and may facilitate presynaptic and postsynaptic actin alignment
Dhanrajan et al., Hippocampus, 2004	Postsynaptic mushroom spine; Emerged from edge of segmented PSD and projected into presynaptic bouton	Hippocampal dentate gyrus of aged (22-month-old) rats	High-frequency stimulation *in vivo* to induce LTP	Optical microcopy and EM; Increased formation of segmented perforated synapses, which in one example bore a spinule	Increase synaptic strength; Participate in learning and LTP
Spacek and Harris, J Neurosci, 2004	Spinules originated from thin and mushroom spine heads, necks, and axons; Engulfed by presynaptic axons, neighboring axons, or astrocytic processes	Hippocampal stratum radiatum of mature rats	Adulthood	Serial section EM; Short vesicular or long vermiform evaginations	Interact with glia; Mechanism for synaptic competition in the mature brain; Retrograde signaling; Membrane remodeling
Stewart et al., Neuroscience, 2005	Postsynaptic spinule-like protrusions projected from the PSD and connected two thorns	Hippocampal CA3 stratum radiatum of adult rats	Spatial training increased thorny excrescence (TE) volume, perforated PSDs, number of thorns, and formation of spinules	EM; Spinule-like protrusions	Increase TE plasticity and spine complexity
Richards et al., Proc Natl Acad Sci, 2005	Postsynaptic spine head protrusions (SHPs) extended to reach presynaptic boutons	Organotypic hippocampal slice cultures of 6-day-old mice	Glutamate was exogenously applied to TTX treated slices to stimulate neuronal activity, inducing SHPs within 8 min with directionality toward glutamate	Time-lapse confocal microscopy and EM; Glutamate-induced SHPs where longer than those that occurred spontaneously after TTX treatment	Meditate contact with nearby boutons for the formation new synapses in response to glutamate in spines not recently activated by presynaptic glutamate
Tao-Cheng et al., Neurosci, 2009	Postsynaptic spinules invaginated into presynaptic terminals	Organotypic hippocampal slice cultures of 7-day-old rats	Exposure to high K^+^ for 0.5–5 min to induce depolarization induced numerous spinules at excitatory and inhibitory synapses, which peaked at ~1 min of treatment	EM; Parallel double-membranes; Length from ~80 to ~500 nm; Spinules were pinch-waisted, tubular, or irregular in shape; Pinch-waisted spinules often had a clathrin coating	Absent in synapses at low levels of activity, but formed and disappeared quickly during sustained synaptic activity; Membrane retrieval during synaptic activity
Ueda and Hayashi, J Neurosci, 2013	Postsynaptic spines	Organotypic hippocampal slice cultures of 6–8-day-old rats	Spinules gradually increased dependent on PIP_3_ during LTP induction by two-photon glutamate uncaging	Two-photon imaging; Filopodia-like; Length of spinules extended quickly, reaching 880 nm after LTP induction, and peaked at 4 min	Retrograde signaling; Formation of new synapses with functional presynaptic boutons for altered synaptic connectivity
Chazeau et al., EMBO J, 2014	Postsynaptic finger-like spine head protrusions (SHPs)	Dissociated hippocampal neuron cultures of rats	Compared F-actin and Arp2/3 movements in mature spines; Cytochalasin D treatment decreased spinule formation	Super-resolution dSTORM/PALM; ~70% of globular or cup shaped spines when imaged at low resolution were composed of several finger-like extensions when imaged by super-resolution	F-actin elongation proteins concentrate at tips of SHPs moving away from PSD; F-actin-driven spine remodeling and motility during structural plasticity
Weinhard et al., Nat Commun, 2018	Postsynaptic sites and dendritic shafts frequently elicited transient spine head filopodia (SHF) from mature spines at microglia contact points	Organotypic hippocampal neuron cultures of 4-day-old C57BL/6J mice	Microglia processes contacted mature, persistent spines, which extended SHFs toward microglia; Some spines relocated to sites of the SHF tips and were more stable than spines without SHFs	Light sheet fluorescence microscopy; Correlative light and electron microscopy (CLEM); Protrusions from spine heads	Microglia-induced spinules mediate synaptic switching and/or stability of mature spines and the formation of new synapses
Zaccard et al., Neuron, 2020	Postsynaptic mushroom spines; Short-lived spinules extended and retracted rapidly, sometimes contacting spine head proximal boutons; Long-lived spinules contacted spine head distal presynaptic boutons and sometimes trafficked PSD fragments to their tips	Dissociated cortical neuron cultures of C57BL/6J mice; Somatosensory cortex from acute brain slices of one-month-old Thy1-YFP-H mice	Acute NMDAR activation to increase neuronal activity enhanced number and length of spinules, which extended to preferentially contact adjacent boutons; Kalirin-7 exogeneous expression increased spinules; Ca^2+^ chelator treatment decreased spinules	Most spinules were dynamic, small (<0.5 μm), short-lived (<60 s), associated with simple PSDs, and variously shaped; Fewer spinules were elongated, stable, long-lived (≥60 s), associated with complex PSDs, and shaped like filopodia, thin spines, or mushroom spines	Small, short-lived spinules explore their environment; Larger long-lived spinules form connections with spine head distal presynaptic terminals during increased activity; Structural plasticity and altered connectivity; Multi-synaptic spines
Campbell et al., eNeuro, 2020	Postsynaptic spines or adjacent boutons and axons; Enveloped by presynaptic boutons and termed spinule-bearing boutons (SBBs)	Primary visual cortex of female and male ferrets	Prevalence of cortical SBBs in V1 increased across postnatal development; ~25% of excitatory boutons in late adolescent ferret V1 contained spinules	EM; Finger-like projections	Mechanism for extrasynaptic neuronal communication; Provide structural “anchors” to increase cortical synapse stability
Gore et al., Front. Synaptic Neurosci, 2022	Adjacent excitatory axons and boutons, postsynaptic dendritic spines, or adjacent non-synaptic spines	Hippocampal CA1 stratum radiatum of adult rats	Adulthood; Ubiquitous at excitatory synapses	FIB-SEM; Thin, finger-like projections; Two subtypes included small clathrin-coated spinules, and larger non-clathrin coated spinules	Small spinules strengthen and stabilize synaptic connections or increase communication *via* trans-endocytosis; Large spinules increase extrasynaptic membrane interface for stability and communication

Recent studies have demonstrated that microglia are essential for normal brain wiring, in addition to their established role in innate responses to immunological stimuli and injury (Eyo and Wu, 2013; Wu et al., 2015; Hong et al., 2016). Microglia survey the local environment in the steady-state by rapidly extending and retracting finger-like membrane processes, which mediate transient contacts with synapses (Tremblay et al., 2011; Eyo and Wu, 2013; Wake et al., 2013). *In vivo* studies of the mouse lateral geniculate nucleus revealed that interactions between microglial processes and extranumerary presynaptic terminals trigger their elimination by phagocytosis, termed “pruning” (Schafer et al., 2012; Stephan et al., 2012). These data support the concept that microglia regulate synapses during early developmental synaptic refinement and experience-dependent plasticity, impacting neural circuit structure and function, as well as cognition and behavior (Stephan et al., 2012; Wu et al., 2015; Hong et al., 2016). However, the underlying molecular mechanisms are still being elucidated and the role of spinules is unclear. One study used EM to characterize microglia-synapse interactions in the visual cortex of juvenile mice and found that microglia processes simultaneously contact synaptic elements at multiple synapses (Tremblay et al., 2010). Furthermore, clathrin-coated pits reminiscent of spinules were observed at the interfaces between microglia and synaptic elements, suggesting they function in cell signaling *via* clathrin-mediated trans-endocytosis of membrane-bound receptors and ligands. In a more recent study, Gross and colleagues used time-lapse, light sheet microscopy to investigate microglia-driven synapse remodeling in developing organotypic hippocampal cultures (Weinhard et al., 2018). Their results showed that microglia frequently contact mature, persistent spines, which subsequently stretch and extend “spine head filopodia” toward the microglia. Roughly one quarter of these spines relocated to the site of the spine head filopodia tip and were significantly more stable than spines without spine head filopodia. In several examples, the spine bearing these spinule-like structures switched to associate with an adjacent axonal bouton. The prevalence of spine head filopodia on microglia-contacted mature spines was confirmed using EM of fixed brain tissue. To a lesser degree, microglia processes also contacted transient spines, which formed *via* filopodia extending from the microglia contact point, or near the dendritic shaft. These data together suggest a role for microglia-induced spinules in synaptic switching and/or stability of mature spines and the formation of new synapses. Additional investigations are needed to assess whether these observations represent a wide-spread phenomenon, and to determine the molecular underpinnings of microglia-mediated spinule induction. Together, published studies suggest a range of spinule functions from retrograde signaling and membrane remodeling to initiation and/or maintenance of synaptic connections.

### 3.2. Spinules in development, adulthood, and aging, and evidence of distinct spinule subsets

Two recent studies investigated spinule prevalence and structure in development and adulthood. In an EM-based investigation of the ferret visual cortex, Nahmani and colleagues assessed spinules enveloped by excitatory presynaptic boutons, termed spinule bearing boutons (SSBs), at key developmental time points (Campbell et al., 2020). Notably, spinules engulfed by boutons typically originated from either postsynaptic spines, or nearby axons and boutons. The prevalence of SBBs increased during postnatal development, with ∼25% of excitatory boutons classified as SSBs in late adolescence. Another recent report utilized focused ion beam scanning EM (FIB-SEM) in the adult male mouse hippocampus to analyze individual synapses by identifying the postsynaptic neurite, the presence of SBBs, origins of spinules within SBBs, and perforated PSDs (Gore et al., 2022). In this study, 74% of excitatory boutons bore spinules and these SBBs were found to be 2.5 times larger than non-SBBs. Spinules within SBBs were often associated with perforated PSDs, an indicator of increased activity, and originated from adjacent axons and boutons, postsynaptic spines, or adjacent non-synaptic spines. Importantly, two subtypes of spinules were detected within SBBs, including smaller clathrin-coated spinules and larger non-clathrin-coated spinules. These results strongly suggest differences in spinule prevalence throughout development and adulthood, as well as the existence of structurally and functionally distinct spinule subsets.

Very few studies have directly investigated the role of spinules in aging. Given that they are generally associated with perforated synapses, leading investigators have proposed that changes in spinules accompany changes in perforated synapses during aging (Petralia et al., 2015). Age-related changes in the prefrontal cortex typically involve loss of thin spines, which are linked to synaptic plasticity and cognitive function (Petralia et al., 2014). On the other hand, age-related changes in the hippocampus often involve loss of large, perforated mushroom spines, which can underly memory and learning (Petralia et al., 2014). For example, perforated synapse loss has been reported in the dentate gyrus of aged, memory-impaired rats (Geinisman et al., 1986). One investigation of the rat parietal cortex assessed perforated synapses at nine ages, ranging from 0.5 to 22 months, from youth to old-age, and non-perforated synapses at 0.5, 12, and 22 months (Jones and Calverley, 1991). In perforated synapses, PSD size and complexity increased with age. At 0.5 and 1 month, perforations tended to be discrete and small with a negatively curved PSD, while small segments of the PSD were sometimes positively curved and associated with small spinules. At 4 months, discrete perforations were larger, with short broad spinules, and this trend continued at 7 and 10 months of age. At 12 months, the majority of PSDs were large, segmented, and associated with large spinules, which projected into presynaptic terminals. Overall, perforated synapses and spinules peaked in middle-aged rats, and these spinules were distinctly larger and projected deeper into presynaptic terminals. These data suggest differences in spinule prevalence and functions in development, adulthood, and aging, as well as the presence of unique spinule subsets.

**Figure 1:**
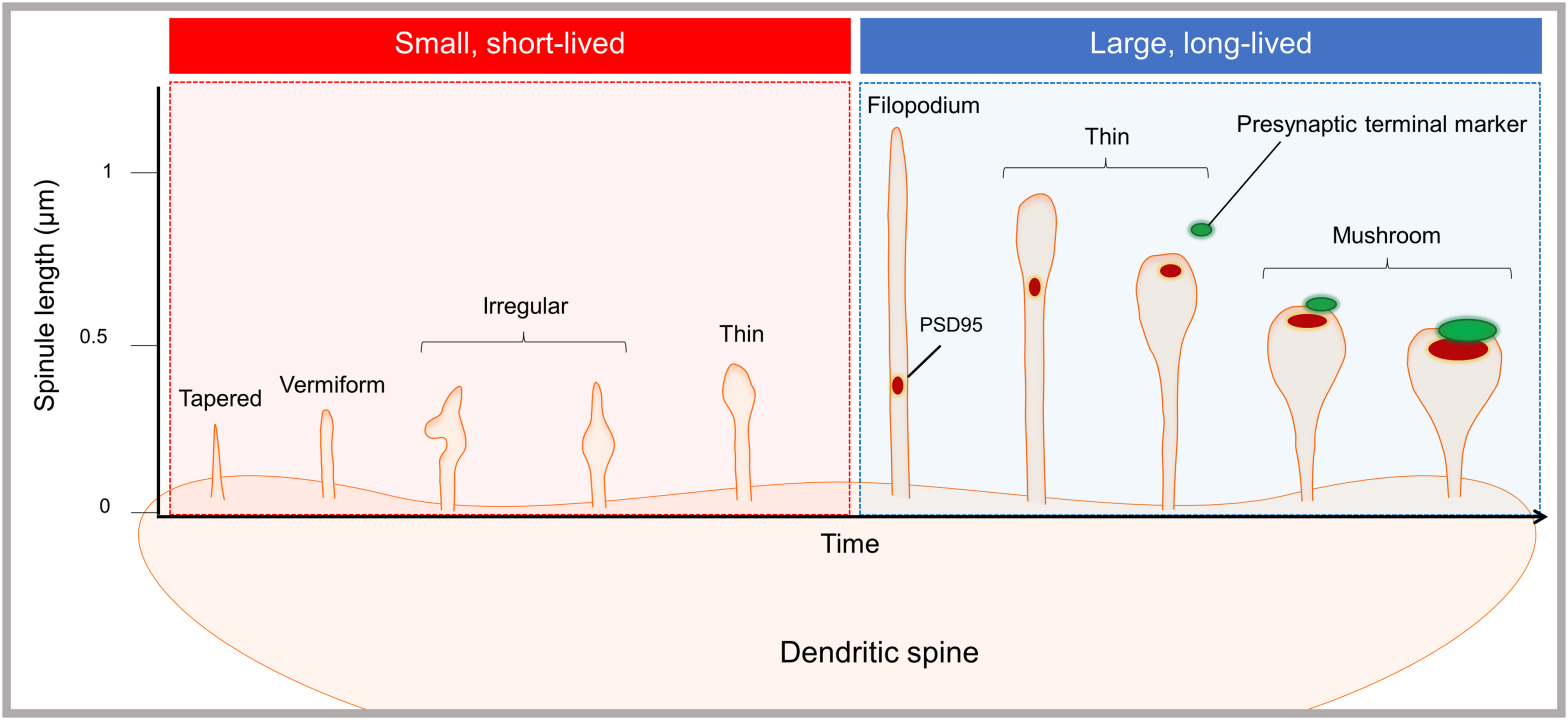
Emergence of morphologically and functionally distinct spinule subsets. The proposed spinule classification system is based on our recent live-cell, time-lapse, enhanced resolution imaging study of spinules in cortical mouse neurons (Zaccard et al., 2020). The majority of spinules emerging from mushroom spines were dynamic and short-lived, existing for sec and up to 1 min, while a minority of spinules were larger and long-lived. Short-lived spinules were typically less than 0.5 microns in length, and displayed diverse morphologies, e.g., tapered, vermiform, irregular, and thin shapes. Long-lived spinules existed for ≥60 s, in some cases exceeding imaging durations up to 10 min, and were typically between 0.5 and 1 microns in length. Additionally, a subset of filopodia-shaped spinules extended up to several microns. Both filopodia and thin spinules often trafficked PSD fragments or contacted presynaptic terminals, but not both. Mushroom spinules were the most stable, with colocalized presynaptic and postsynaptic markers at enlarged spinule tips. Synaptic activity enhanced the number of spinules and the proportion of elongated, long-lived spinules.

Our recent, time-lapse rapid SIM and enhanced resolution confocal imaging study of dissociated cortical mouse neurons supports the existence of at least two functionally and morphologically distinct spinule subsets (Zaccard et al., 2020). In the basal state, most spinules emerging from mushroom spines were dynamic and short-lived (<60 s), extending and retracting repeatedly at the same topographical spine head locations, while subset of elongated spinules was more stable and long-lived (≥60 s). Short-lived spinules typically emerged from the edges of simple PSDs, while long-lived spinules were associated with more complex PSDs. Exclusively in long-lived spinules, we often observed PSD95 fragments trafficking to spinule tips and Ca^2+^ nanodomains, in synchrony with, but isolated from spine head Ca^2+^ transients. Additionally, neuronal activity increased the number and length of spinules, and elongated spinules preferentially contacted adjacent boutons, which were not presynaptic to the spine head. Long-lived mushroom-shaped spinules sustained dynamic interactions with large spine head distal presynaptic terminals, and PSD95 puncta colocalized with presynaptic markers at the bulbous spinule tips. We confirmed our findings of dynamic, short-lived spinules and elongated, stable, long-lived spinules in the somatosensory cortex in acute mouse brain slices from one-month-old mice. We propose that spinule morphology and dynamics are on a continuum echoing that of spine classes (Figure 1). Long-lived filopodia- and thin-shaped spinules can traffic PSD fragments or interact with distal presynaptic terminals, suggesting their role in new synapse formation, while mushroom spinules mediate sustained contact with presynaptic terminals. These data together suggest that long-lived spinules can facilitate the formation of secondary synapses, potentially resulting in multi-contact synapse formation and altered connectivity.

**Figure 2:**
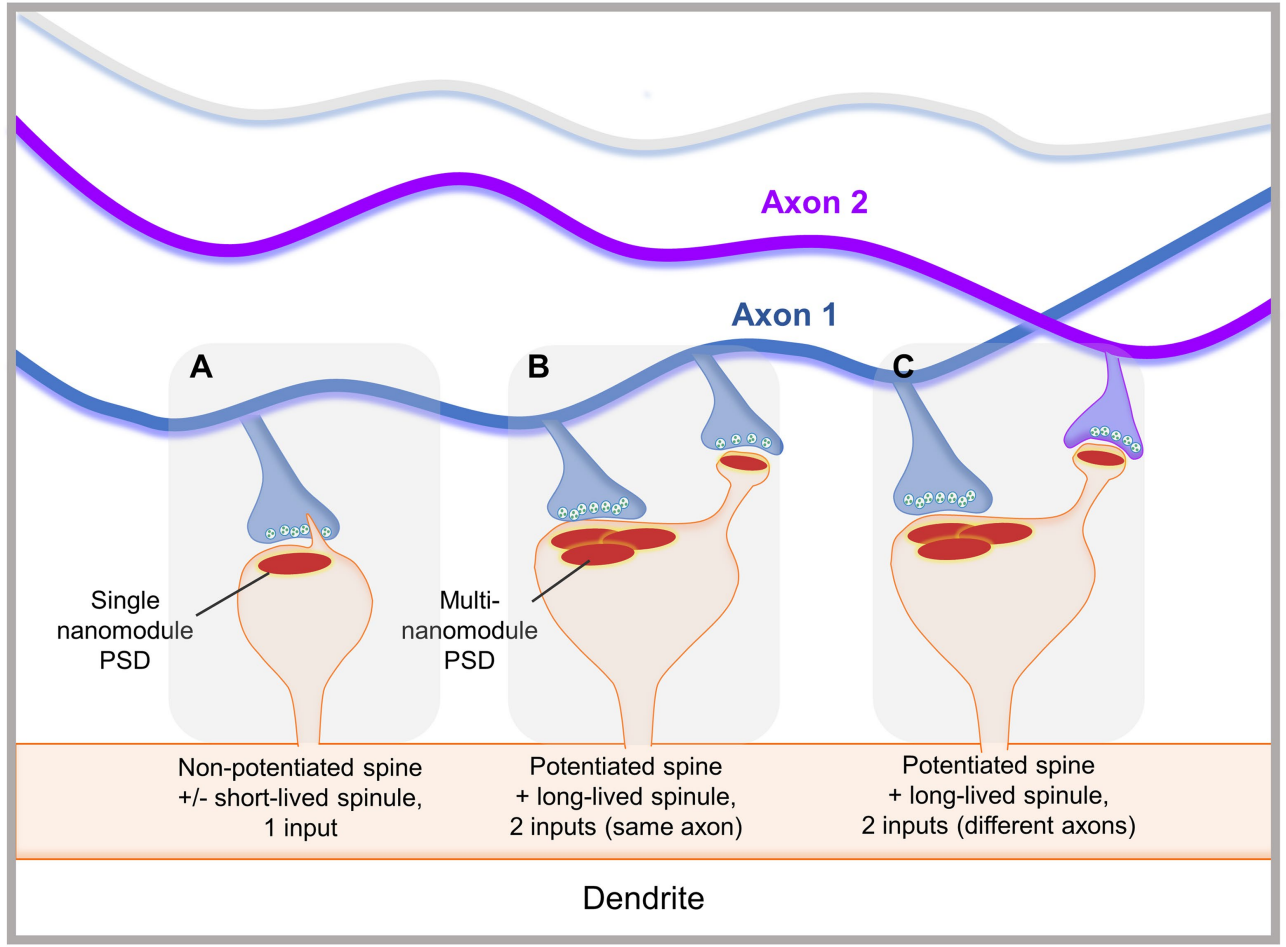
Proposed functions of spinules in mediating localized structural synaptic plasticity. **(A)** A smaller, non-potentiated spine with a simple PSD occasionally forms small, dynamic short-lived spinules, which emerge from close to the PSD edge. Small spinules may be trans-endocytosed or explore their environment. **(B)** A large, potentiated spine displays a complex PSD, with multiple nanomodules, and an elongated long-lived spinule. The spinule forms a secondary synaptic connection with a bouton from the same axon as the one forming a synapse with the spine head. **(C)** A large, potentiated spine with multiple PSD nanomodules displays a long-lived spinule that contacts an adjacent bouton originating from a different axon than the one synapsing with the spine head. These interactions may facilitate secondary synapse formation, potentially leading to alterations in synaptic plasticity, connectivity, and down-stream brain function.

These studies collectively suggest the presence of spinule subsets that are divergent in structure, dynamics, and function. Small, clathrin-coated spinules observed by EM may be involved in clathrin-mediated trans-endocytosis, while large non-coated spinules may be involved in forming and/or maintaining complex synapses. We propose that small, short-lived spinules mediate communication with presynaptic partners or glia *via* trans-endocytosis, while elongated long-lived spinules facilitate the formation, stabilization, or modification of synaptic connections (Figure 2). Additionally, some small spinules may serve as precursors to larger, elongated spinules, which are enhanced by synaptic activity. The synapse has long been presented as a one-to-one presynaptic and postsynaptic connection. However, “multi-synaptic boutons,” wherein multiple spines contact one presynaptic terminal to form one-to-many connections, have been reported in the hippocampus, cortex, and cerebellum in association with sensory experience, brain lesions, and learning (Yang et al., 2018). Additionally, “multi-synaptic spines,” which receive inputs from multiple presynaptic boutons to form many-to-one connections, have been observed in complex motor learning in rats, fear conditioning in mice, and epilepsy (Jones et al., 1999; Kuwajima et al., 2013; Yang et al., 2016). We hypothesize that contact between spinules and distal presynaptic terminals can lead to the formation of secondary synapses and multi-synaptic spines, providing an additional modality for altering dendritic spine connectivity (Figure 2). Hence, spinules can represent a mode of structural synaptic plasticity that can modulate pre-existing synapse structure and downstream function or facilitate the formation of connectivity-altering secondary synapses.

## 4. Spinules in psychiatric disorders

Structural synaptic plasticity is important for key neurological functions, such as memory and cognition, and aberrant spine dynamics have been associated with many psychiatric disorders. *In vivo* studies have demonstrated that spines are altered in response to environmental factors impacting brain function. For example, visual stimulation increases spine density in the rat visual cortex, while visual deprivation results in abnormal spine shape and reduced spine number in the rabbit visual cortex (Hering and Sheng, 2001). Additionally, spines are typically found to be abnormally small and immature in human patients and most animal models of intellectual disability (Ramakers, 2002; Kasai et al., 2010). We propose that pathological changes in spines can include not only differences in size, shape, and density, but also differences in spinule formation. To date, a limited number of published studies have reported on the presence or absence of spinules in psychiatric disorders. Herein, we review those studies noting differences in spinules in human patients and disease models associated with altered cognitive function and memory (Table 2). The current literature suggests a link between decreased spinules and impaired synaptic plasticity and/or intellectual disability and between increased spinules and hyper-excitability and/or neurodegenerative diseases.

**Table 2:** Spinule structure and proposed functions in psychiatric disease.

Author, journal, year	Spinule origination and contacts	Cell type and brain region	Human disease or disease model	Microscopy technique, spinule prevalence, and spinule morphology	Proposed spinule functions
Anglade et al., Neurodegeneration, 1996	Postsynaptic spinules originated from spines with perforated PSDs and invaginated afferent terminals of asymmetric synapses	Subcortical caudate nucleus	Parkinson’s disease (PD) versus (vs.) age matched controls	EM; Invaginations resembling spinules more pronounced between two postsynaptic densities	Altered synaptic plasticity and transmission in corticostriatal synapses of PD patients in response to hyperactivity
Sasaki and Iwata, Neurosci Lett, 1999	Not reported; Degenerating presynaptic terminals often displayed spinule-like structures, in one case from the somata	Normal appearing Betz cells in the 5th layer of motor cortex	Amyotrophic lateral sclerosis (ALS) patients vs. age-matched controls	EM; Presynaptic terminals often displayed dark presynaptic vesicles and mitochondria, absent or obscure postsynaptic membranes, and increased spinules in ALS patients	Synaptic alteration in normal-appearing Betz cells in ALS
Zhang and Houser, J Comp Neurol, 1999	Postsynaptic spinules originated from spines with perforated PSDs and protruded into reorganized inner molecular layer presynaptic mossy fiber terminals	Granule cells of the hippocampal dentate gyrus	Temporal lobe epilepsy (TLE) patients	EM; Increased number of spinules extended from perforations in the PSD; Some spinules were elongated and extended into adjacent mossy fiber terminals	Active perforated synapse remodeling and/or multi-synaptic bouton formation to increase granule cell hyperexcitability in TLE; May provide diffusion barrier and increase formation of independent active zones
Popov et al., J Comp Neurol, 2010	Postsynaptic spinules originated from large and complex spines, termed thorny excrescences (TEs)	Hippocampal dentate gyrus and area CA3	Ts65Dn mouse model of Down Syndrome (DS) vs. 2N mice	EM; Decrease in spinule-like protrusions from TEs in Ts65Dn mice	Decreased synaptic plasticity, reduced TE connectivity, synaptic transmission, and down-stream cognitive and memory function in DS
Blanque et al., Front Neuroanat, 2015	Postsynaptic spines	Neocortical and hippocampal neurons	KIBRA knock-out (KO) mice; KIBRA gene has been linked to human memory performance	In KIBRA KO mice, reduced double plasma membrane structures, <0.3 μm in diameter, surrounded by ≥3 synaptic vesicles	Decreased perforated synapses and spinules in KIBRA KO neurons may contribute to impaired long-term synaptic plasticity
Steward et al., Front Mol Neurosci, 2021	Postsynaptic spinules emerged from complex spines with enlarged heads and segmented PSDs and extended into non-degenerating presynaptic terminals	Granule cells of the hippocampal dentate gyrus	C57Bl/6 mice with Wld^s^ mutation, which delays Wallerian degeneration onset vs. wildtype mice, after orphaned axon induction by surgical entorhinal cortex lesion	EM; In Wld^s^ mutant mice, some enlarged spine heads contacting non-degenerating orphaned presynaptic terminals displayed finger-like protrusions	Trans-synaptic signaling processes between orphaned axons and postsynaptic targets to delay axonal degeneration; Multi-synaptic bouton formation

### 4.1. Decreased spinules are associated with impaired synaptic plasticity and intellectual disability

The single nucleotide polymorphism in the KIBRA (KIdney/BRAin) gene has been linked to individual differences in human memory performance, which become more prominent in aging (Witte et al., 2016). Additionally, deletion of the gene encoding the KIBRA protein results in impaired long-term synaptic plasticity (Papassotiropoulos et al., 2006; Makuch et al., 2011). In an EM study of the neocortex and hippocampus in KIBRA knock-out mice, researchers found that synapse ultrastructure and density were generally normal, but they displayed fewer spinules and perforations (Blanque et al., 2015). Another EM study examined the synaptic ultrastructure of the hippocampus dentate gyrus and area CA3 in the Ts65Dn mouse model for Down Syndrome (DS), which is associated with learning and memory deficits (Popov et al., 2011). Large and complex spines, termed thorny excrescences (TEs), receive inputs from dentate gyrus mossy fibers, and 3D reconstructions revealed a reduced number and volume of TEs, as well as a decrease in spinule-like protrusions. These morphological changes of TEs in Ts65Dn mice suggest a role for spinules in TE connectivity, synaptic transmission, and down-stream cognitive and memory function. While the presence of spinules in human DS models is not well documented, abnormal dendritic spine structure and function is one of the most prominent features of the disease and is directly linked to intellectual disability (Torres et al., 2018). Thus, these studies together suggest that a decrease in spinules may represent a structural correlate for reduced synaptic plasticity and/or diseases of intellectual disability.

### 4.2. Increased spinules are associated with hyperexcitability and neurodegenerative diseases

Temporal lobe epilepsy (TLE) is a common localized epilepsy characterized by complex partial seizures, epileptic discharges stemming from temporal lobe limbic structures, and hippocampal neuron loss and gliosis (Sutula, 1990). Humans with medically intractable TLE typically display loss of hippocampal neurons and reorganization of granule cell axons and their collaterals, termed mossy fibers, to the inner molecular layer of the dentate gyrus (Zhang and Houser, 1999). This “sprouting” of mossy fibers often precedes the onset of seizures in animal models and may contribute to increased spontaneous seizure susceptibility observed in TLE (Tauck and Nadler, 1985; Ben-Ari and Represa, 1990; Babb et al., 1992; Buckmaster and Dudek, 1997). An examination of mossy fiber ultrastructure in TLE patients revealed that mossy fiber terminals contact postsynaptic spines, often forming complex, multi-spine connections (Zhang and Houser, 1999). In some cases, postsynaptic spines displayed perforations and spinules, which were sometimes elongated and protruded into mossy fiber terminals. These results suggest an abundance of highly active synapses in the inner molecular layer of the dentate gyrus in TLE. Additionally, structural changes in mossy fiber connections, including spinules, could play a significant role in multi-synaptic bouton formation and granule cell hyperexcitability in TLE.

Parkinson’s disease (PD) is the second most common age-related neurodegenerative disease and is associated with cognitive and memory impairment (Aarsland et al., 2021). In an EM study of asymmetrical synapses in the caudate nucleus of PD patients, dendritic spines displayed increased PSD length and number of perforated PSDs, suggesting hyperactivity, as well as increased spinule-like structures, compared to age-matched controls (Anglade et al., 1996). Importantly, the size and density of spines and the size of PSD perforations were unchanged. These results suggest that dynamic changes in spinules may contribute to altered synaptic plasticity and transmission in the corticostriatal synapses of PD patients in response to hyperactivity. Researchers investigated synaptic plasticity in Betz cells in an EM study of the motor cortex of amyotrophic lateral sclerosis (ALS) patients (Sasaki and Iwata, 1999). Presynaptic terminals displayed increased spinule-like structures, accompanied by a range of changes indicative of hyperexcitability and neurodegeneration, such as aggregated presynaptic vesicles and increased mitochondria (Sasaki and Iwata, 1999; Kann and Kovacs, 2007). These studies suggest that increased spinules and changes in spinule dynamics may be a feature of synaptic changes in hyperexcitability and progressive neurodegenerative disorders. However, the role of spinules in neuronal hyperactivity and in altered synaptic transmission and plasticity underlying neurodegenerative diseases requires further investigation.

Research on axon degradation has largely focused on experimental models of Wallerian degeneration, a process in which axonal injury at a defined site and time simultaneously affects all axons in a nerve, leading to sensory and motor deficits (Conforti et al., 2014). Originally arising spontaneously in C57Bl/6 mice, the Wallerian degeneration slow (Wld^s^) gene mutation reverses this process, prolonging the viability of “orphaned axons,” i.e., axons that survive separated from their parental cell body, to delay the onset of Wallerian degeneration (Lunn et al., 1989; Perry et al., 1990). A recent EM study of the Wld^s^ mutation examined spines in granule cells of the hippocampal dentate gyrus following lesions of perforant path inputs from the entorhinal cortex (Steward et al., 2021). Spines contacted by orphaned axons displayed drastic morphological changes, including hypertrophy of spine heads, enlargement of PSDs, and the formation of spinules extending into presynaptic terminals. While the signaling mechanisms between the postsynaptic neuron and orphaned axons are largely unknown, the observed increased spine complexity and spinules was similar to that seen following synaptic activity and induction of LTP (Petralia et al., 2015). These results suggest one possible future direction to explore the role of spinules in facilitating trans-synaptic signaling between orphaned axons and their postsynaptic targets to delay axonal degeneration.

## 5. Discussion

A growing body of evidence demonstrates that spinules are a feature of increased neuronal activity and LTP and suggests their function in mediating localized structural synaptic plasticity. Mechanistic studies have revealed that PIP_3_, local Ca^2+^ signaling, and kalirin-7 are involved in the regulation of spinules. However, the field could benefit from additional studies focused on connecting the known molecular players and further delineating the molecular pathways underlying spinule formation. Multiple studies suggest that spinules can participate in cell signaling *via* trans-endocytosis, or mediate formation of new synapses and maintenance of stable complex synapses. Additionally, microglia-driven contacts with spines can induce spinule formation potentially to facilitate synaptic switching, stability, or complexity of mature spines, and to form new synapses at sites of transient spines (Weinhard et al., 2018). A few key studies have provided insights into the prevalence and possible functions of spinules in development, adulthood, and aging. One developmental study showed that spinules peak in late adolescence, implicating spinules in synaptic plasticity during key periods of brain development (Campbell et al., 2020). In general, the literature suggests that spinule number and size increase with age, peaking in middle-age, and that spinules are a prominent feature of complex, active synapses in adulthood. We propose that spinules mediate structural plasticity during key developmental periods and, most prominently, structural plasticity of complex spines in adulthood, followed by a slow decline of spinules in aging. Future studies would be beneficial for further delineating the structure and functions of spinules in development, adulthood, and aging.

Recent advances in microscopy techniques have enabled live, time-lapse imaging studies of spinules to enhance our current knowledge of their structure and function. Importantly, standard light microcopy techniques cannot adequately detect smaller spinules. Electron microscopy provides the necessary resolution, but captures only frozen snap-shots in time, yielding little information about spinule dynamics. Newer studies provide convincing evidence of structurally and functionally distinct spinule subsets (Zaccard et al., 2020; Gore et al., 2022). In general, spinule shape, dynamics, and functions may echo that of dendritic spine classes. Small, dynamic, short-lived spinules are associated with simple PSDs and may serve to explore their environment or mediate retrograde signaling and/or membrane remodeling *via* trans-endocytosis. Larger, elongated, long-lived spinules are associated with complex PSDs, are enhanced by activity, and preferentially contact spine head adjacent boutons as opposed to the spine’s presynaptic partner. These data implicate large, long-lived spinules in altering synaptic connectivity and stability, including the formation of multi-contact synapses. Hence, distinct spinule subtypes likely mediate structural synaptic plasticity, which is known to underlie cognition, memory, and many psychiatric disorders, *via* divergent mechanisms. Additional investigations are needed to further advance our understanding of spinule structural and functional diversity in health and psychiatric disease.

While spinules remain understudied in the context of disease, a number of investigations have reported changes in spinules in psychiatric disorders. Generally, decreased spinules are associated with impaired synaptic plasticity and intellectual disability, while increased spinules accompany hyperexcitability and neurodegenerative disease. The main caveat to these inferences remains the limited number of published studies that have reported the presence or absence of spinules in human disease. Hence, additional studies are necessary to delineate the molecular mechanisms underlying spinule dysfunction and determine the role of spinules in development, adulthood, aging, and psychiatric disorders. An improved understanding of spinule regulation, dynamics, function, and dysfunction could reveal spinules as therapeutic targets, or as markers for diseases that involve altered structural synaptic plasticity.

## Author contributions

PP and CG: project supervision. CZ, IG, and AS: conceptualization and manuscript preparation and editing. All authors contributed to the article and approved the submitted version.

## Funding

This study was supported by the NIH grants R01MH107182, 2R56MH071316-16, and P30AG072977.

## Conflict of interest

The authors declare that the research was conducted in the absence of any commercial or financial relationships that could be construed as a potential conflict of interest.

## Publisher’s note

All claims expressed in this article are solely those of the authors and do not necessarily represent those of their affiliated organizations, or those of the publisher, the editors and the reviewers. Any product that may be evaluated in this article, or claim that may be made by its manufacturer, is not guaranteed or endorsed by the publisher.
